# Dynamic Load Identification of Thin-Walled Cabin Based on CNN-LSTM-SA Neural Network

**DOI:** 10.3390/ma18061255

**Published:** 2025-03-12

**Authors:** Jun Wang, Shaowei Song, Chang Liu, Yali Zhao

**Affiliations:** 1 National Key Laboratory of Aerospace Liquid Propulsion, Xi’an Aerospace Propulsion Institute, Xi’an 710100, China; junw83@163.com (J.W.); songshw82@163.com (S.S.); 13720753215@163.com (C.L.); 2 School of Aerospace Engineering, Xi’an Jiaotong University, Xi’an 710049, China; 3 State Key Laboratory for Strength and Vibration of Mechanical Structures, School of Mechanical Engineering, Xi’an Jiaotong University, Xi’an 710049, China

**Keywords:** load identification, CNN-LSTM-SA, thin-walled compartment, time sequence mapping

## Abstract

Spacecraft are subjected to various external loads during flight, and these loads have a direct impact on the structural safety and functional stability of the spacecraft. Obtaining external load information can provide reliable support for spacecraft health detection and fault warning, so accurate load identification is very important for spacecraft. Compared with the traditional time-domain load identification method, the neural network-based time-domain load identification method can avoid the establishment of the inverse model and realize the response-load time-sequence mapping, which has a broad application prospect. In this paper, a CNN-LSTM-SA neural network-based load identification method is proposed for load acquisition of a thin-walled spacecraft model. Simulation results show that the method has higher identification accuracy and robustness (RMSE and MAE of 8.47 and 10.83, respectively, at a 20% noise level) in the load identification task compared to other network structures. The experimental results show that the coefficients of determination (R^2^) of the proposed neural network load recognition model for time-domain identification tasks of sinusoidal and random loads are 0.98 and 0.93, respectively, indicating excellent fitting performance. This study provides a reliable new method for load identification in thin-walled spacecraft cabin structures.

## 1. Introduction

In the field of aerospace, the acquisition of external load information is directly related to the stability and reliability of spacecraft. However, due to the harsh external environment and complex dynamic loads during spacecraft flight, it is difficult to measure external loads directly with sensors, so it is necessary to use load identification techniques to estimate external loads from the response inside the spacecraft [1]. The concept of load identification originated in the 1970s. At that time, load identification techniques were used to determine actual aircraft loads in order to improve aircraft performance [2]. It has since been widely applied in various engineering fields due to its ability to improve product reliability and durability. Dynamic load identification is divided into two categories: time-domain load identification methods and frequency-domain load identification methods. Time-domain load identification technology does not need to consider the frequency characteristics of the structure and is suitable for dynamic problems with significant nonlinearity and uncertainty. At the same time, the time-domain method can realize the on-line monitoring of structural loads, so the research on time-domain load identification methods has received more and more attention in recent years [3].

Time-domain load identification usually relies on the Green’s kernel function deconvolution method (GKFM) [4], which is mainly for linear systems. Meanwhile, in order to solve the matrix ill-posedness problem, the regularization method is needed in the solution process. However, the selection of the regularization parameters is a more complicated task [5]. With the development of deep learning, neural networks are gradually being applied to the task of load identification. Compared with the traditional time-domain load identification methods, the neural network-based method has the ability of automatic feature extraction and powerful nonlinear modeling capability, as well as better robustness [6]. Luis et al. [7] used a neural network to achieve the identification of horizontal and vertical forces on automobile wheel hubs and discussed the potential of the method for online monitoring of loads applied to automobiles. Tang et al. [8] proposed a method combining stochastic response power spectral density and deep convolutional neural network (CNN) to accurately recognize vehicle load information. Zhang et al. [9]. proposed a CNN network based on a transform domain approach, using decomposed signals from wavelet transforms of multiple vibration signals as input; combined with a CNN network to achieve load identification in the time domain. Zhou et al. [10] performed impact load identification for nonlinear structures using a deep recurrent neural network consisting of two long short-term memory network (LSTM) layers and one bi-directional long short-term memory network (BiLSTM) layer; the network trained on numerous dynamic responses and impact loads demonstrated the ability to identify complex impact loads, even when the impact location was unknown. Yang et al. [11] proposed a neural network model with a bi-directional LSTM layer, an LSTM layer, and two fully connected layers to identify typical dynamic loads (sinusoidal, impact, and random loads) for simply supported beams. Furthermore, Yang et al. [12] developed a depth-expanded convolutional neural network, which directly constructs a transfer model between the structural acceleration response and excitation for data-driven dynamic load identification. Wang et al. [13] proposed a deep regression adaptive network based on model migration that learns to improve the accuracy and efficiency of neural networks for load identification.

Due to the different characteristics of different neural networks, recent research in time-series data prediction has focused on combining multiple networks, thereby leveraging their strengths and building more accurate and efficient models. Lu et al. [14] proposed a CNN-BiLSTM-AM (attention mechanism) neural network for predicting stock data, which confirms the significant advantages of this method over other neural network methods. Huy et al. [15] used a CNN-LSTM model combined with an attention mechanism to predict short-term power loads, addressing issues with input–output relationships, mitigating information loss from long input time-series data and improving prediction accuracy. Marios [16] examined dynamic structural loads of gated cyclic units, using an LSTM and CNN trained on small datasets, and compared the results with a physically based residual Kalman filter (RKF). Zhang et al. [17] proposed a method to predict wave height based on a CNN-LSTM neural network, and the results show that the method can effectively improve the prediction accuracy as well as robustness. Hu et al. [18] proposed a fall detection method based on a CNN-LSTM neural network and compared the CNN network and LSTM network alone, and the results showed that the network can significantly improve the accuracy of detection. Divya et al. [19] proposed a new hybrid deep learning method SSA (Singular Spectrum Analysis)-CNN-LSTM, which demonstrated the superior performance of this network for solar power generation prediction over a long time period in the future. Numerous studies have shown that higher prediction accuracy as well as robustness can usually be achieved by employing hybrid neural networks in time-series tasks. Whereas most of the related studies are currently focused on time-series prediction tasks, there are fewer related studies on time-series mapping tasks similar to load identification.

In order to further improve the identification accuracy and applicability of neural networks in load identification tasks, and to promote the engineering application of neural network load identification models, this paper proposes a load identification model based on a CNN-LSTM-SA (self-attention mechanism) hybrid neural network. The CNN layer performs feature extraction on the time-series data, the LSTM layer captures the long-term dependencies in the time series, and the SA layer establishes the global dependencies of the time series and assigns different weights. In this paper, the initial time-series data are segmented and used as inputs to the neural network for network training, the load identification accuracy of this neural network is compared to other network models, and its noise immunity is discussed. Finally, experimental studies are conducted on the identification of sinusoidal and random load time-domain information using the method to prove its value for engineering applications.

## 2. CNN-LSTM-SA Neural Network Load Identification Model Building

### 2.1. Load–Response Relationship in the Time Domain

For a linear system, the response induced by the load in the time domain satisfies the principle of linear superposition, and the time-domain response data R(t) and the time-domain load F(t) at the response measurement point satisfy the following Equation [20]:

(1) $$R(t)=\int_{0}^{t}F(t)h(t-\tau )d\tau$$

where R(t) denotes the dynamic response of the system measurement points, e.g., strain, stress, displacement, velocity, acceleration, etc. h(t) is the response point–excitation point impulse response function. By discretizing the continuous time domain into a number of discrete time periods ∆t, Equation (1) can be expressed as follows:

(2) $$\left[\begin{array}{c} R_{1}\\ R_{2}\\ \vdots \\ R_{n} \end{array}\right]=\left[\begin{array}{cccc} h_{1} & 0 & \ldots & 0\\ h_{2} & h_{1} & \ldots & 0\\ \vdots & \vdots & \ddots & \vdots \\ h_{n} & h_{n-1} & \ldots & h_{1} \end{array}\right]\left[\begin{array}{c} F_{0}\\ F_{1}\\ \vdots \\ F_{n-1} \end{array}\right]\Delta t$$

That is to say, for time-series data with time sampling length, the relationship between response and loading satisfies Equation (3). From Equation (3), it can be seen that the response r(t) of the structure at the moment t is not only affected by the current moment f(t) but also by all previous moments f(0)~f(t−1). In other words, the response at any moment t contains all the information about the loads between the moments 0~t. For the inverse of the above process, the load f(t) at the moment t can be written as Equation (4).

(3) $$\left\{\begin{array}{c} r(1)\\ r(2)\\ \vdots \\ r(k) \end{array}\right\}=\left[\begin{array}{cccc} h(1)\Delta t & & & \\ h(2)\Delta t & h(1)\Delta t & & \\ \vdots & \vdots & \ddots & \\ h(k)\Delta t & h(k-1)\Delta t & \cdots & h(1)\Delta t \end{array}\right]\left\{\begin{array}{c} f(0)\\ f(1)\\ \vdots \\ f(k-1) \end{array}\right\}$$

(4) $$f(t)=G\left(r(t),r(t+\Delta t),\cdots ,r(t+n\Delta t)\right)$$

### 2.2. CNN-LSTM-SA Network Architecture

**Figure 1:**
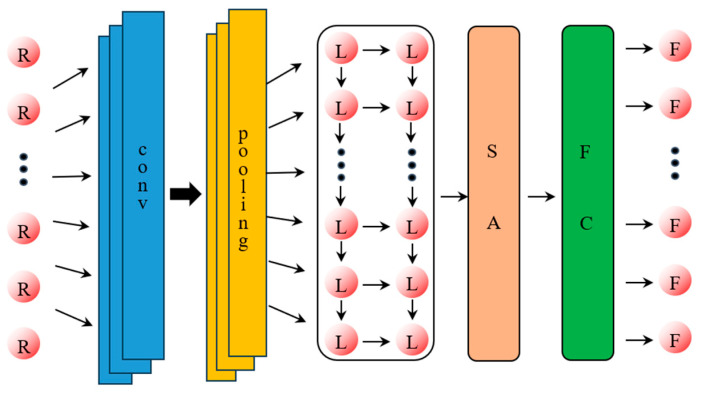
CNN-LSTM-SA load identification model.

The CNN model has excellent feature extraction ability and is widely used in various classification problems. The LSTM model has a special gate structure and weight sharing mechanism, which can avoid losing the long-term features of time-series data and is widely used in time-series analysis. The SA model has the importance of adding the past feature state of the time-series data to the output results and is widely used in adjusting the prediction results from an LSTM model. In this paper, according to the characteristics of the CNN, LSTM, and SA models, a load identification model based on a CNN-LSTM-SA architecture is established, and the structure of the model is shown in Figure 1. The main structure of the model consists of an input layer, CNN layer (one-dimensional convolutional layer and maximum pooling layer), LSTM layer, SA layer, fully connected layer, and output layer.

**Figure 2:**
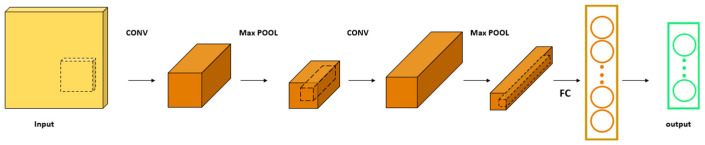
CNN network model (The dashed squares are convolution kernels and the circles indicate the flattened data).

CNN is a feed-forward neural network with good performance in digital image processing, image denoising [21], and classification problems [22]. Its main structure includes a convolutional layer, pooling layer, fully connected layer, and output layer, as shown in Figure 2. The convolutional layer is the core of the CNN, which performs convolutional operations on input data by convolutional kernels of specific sizes. Each convolutional layer contains multiple convolutional kernels, which can extract high-dimensional features from the data and establish the relationship between input and output units through sparse connections. Its unique weight sharing feature allows all output units to share a common set of parameters to connect with the input units, which greatly reduces the training parameters required for the model and saves training time. The pooling layer is used to downsize the data, reducing redundant information while retaining key information and reducing the number of parameters required by the network. The calculational procedure of the convolutional layer is shown in Equation (5):

(5) $$y_{t}=f\left(x_{t}*k_{t}+b_{t}\right)$$

where $x_{t}$ is the input, $k_{t}$ is the weight of the convolution kernel, $b_{t}$ is the bias, f is the activation function, and $y_{t}$ is the output.

**Figure 3:**
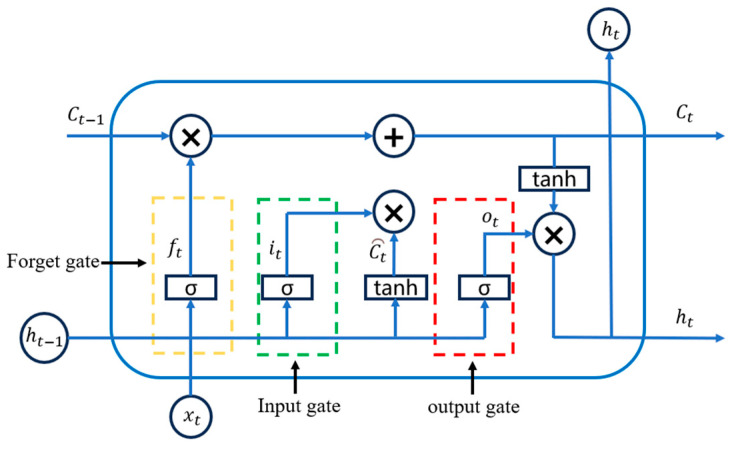
LSTM network memory cell.

The LSTM neural network is a special type of recurrent neural network which is especially used for processing and predicting temporal data [23]. Compared with a recurrent neural network (RNN), a LSTM solves the problems of gradient vanishing and gradient explosion through a special network structure, which is especially suitable for tasks involving long-term dependencies. The memory cell of an LSTM is shown in Figure 3, which controls the flow of data through a “gating mechanism” to selectively remember or forget information to preserve the long-term dependencies of the temporal data. The memory cell of the LSTM consists of three gates (forget gate, input gate, and output gate), each consisting of a sigmoid activation function and a point multiplication operation with values between 0 and 1, which are used to control the flow of data. In Figure 3, $C_{t-1}$ is the state of the cell at the previous moment, $h_{t-1}$ is the final output value of the LSTM cell at the previous moment, $f_{t}$ is the output value of the forget gate, $i_{t}$ is the input of the current input gate, $\overset{⏜}{C_{t}}$ is the temporary state of the current cell, $o_{t}$ is the output value of the output gate, $C_{t}$ is the state of the cell at the current moment, and $h_{t}$ is the final output value of the current cell. The computational procedure of an LSTM is as follows:

(1) The forget gate generates an output value $f_{t}$ between 0 and 1 by reading the final output $h_{t-1}$ of the previous moment and the input $x_{t}$ of the current moment and using Equation (6), where 1 represents the complete retention of information and 0 represents the complete discarding of information.
  (6) $$f_{t}=\sigma \left(W_{f}\cdot \left[h_{t-1},x_{t}\right]+b_{f}\right)$$
  where $W_{f}$ is the weight matrix of the forget gate, $b_{f}$ is the bias term, and σ is the Sigmoid activation function;
(2) The input gate generates the temporary state of the cell at the current moment $\overset{⏜}{C_{t}}$ by reading the final output of the previous moment $h_{t-1}$ and the input of the current moment $x_{t}$ and then updates the cell state in conjunction with the output of the forget gate to obtain the new cell state $C_{t}$ which taking the values from 0 to 1.
  (7) $$i_{t}=\sigma \left(W_{i}\cdot \left[h_{t-1},x_{t}\right]+b_{i}\right)$$
  (8) $$\overset{⏜}{C_{t}}=\tanh\left(W_{c}\cdot \left[h_{t-1},x_{t}\right]+b_{c}\right)$$
  (9) $$C_{t}=f_{t}*C_{t-1}+i_{t}*\;\overset{⏜}{C_{t}}$$
  where $i_{t}$ is the output value of the input gate at the current moment, which determines the extent to which the current input $x_{t}$ affects the update of the unit state $C_{t}$. $W_{c}$ and $b_{c}$ are the weight matrix and bias terms for computing the temporary unit state $\overset{⏜}{C_{t}}$. tanh is the hyperbolic tangent activation function and * denotes the matrix element-by-element multiplication;
(3) The output gate extracts and outputs key information from the current unit state. It also reads the final output value $h_{t-1}$ of the cell at the previous moment and the input value $x_{t}$ of the cell at the current moment and calculates $o_{t}$ through Equation (10).
  (10) $$o_{t}=\sigma \left(W_{o}\left[h_{t-1},x_{t}\right]+b_{o}\right)$$
  where $o_{t}$ takes values from 0 to 1, $W_{o}$ is the weight of the output gate, and $b_{o}$ is the bias of the output gate;
(4) Finally, the final output of the unit at the current moment is calculated from the output value of the output gate $o_{t}$ and the current state of the unit $C_{t}$ by Equation (11).
  (11) $$h_{t}=o_{t}*\tanh\left(C_{t}\right)$$

SA (self-attention mechanism) is a technique that can dynamically capture the dependencies between different positions in a sequence and is widely used in natural language processing, computer vision, and time-series prediction. Its core idea is to decide how to summarize information by calculating the correlation weights of each element in a sequence with other elements. This mechanism allows the model to model the backward and forward relationships of the input sequences on a global scale, effectively capturing long-term dependencies. The main computational procedure for SA is as follows:

The input sequence X is mapped to the Query (*Q*), Key (*K*), and Value (*V*) for representing the characteristics of the current element, the importance of other elements, and the information to be summarized, respectively, through three sets of learnable linear transformations as in Equation (12).

(12) $$Q=XW_{Q},\;K=XW_{K},\;V=XW_{V}$$

where $W_{Q}$, $W_{K}$, and $W_{V}$ are trainable weight matrices.

The similarity score is obtained by computing the dot product of the Query and Key as in Equation (13), which is usually divided by $\sqrt{d_{k}}$ in order to balance the numerical stability, where $d_{k}$ is the Key dimension:

(13) $$Similarity_{i,j}=\frac{Q_{i}\cdot K_{j}^{T}}{\sqrt{d_{k}}}$$

Use the softmax function to normalize the scores to a *probability* distribution:

(14) $$\alpha_{i,j}=\mathrm{softmax}\left({Similarity}_{i,j}\right)=\frac{\exp\left({Similarity}_{i,j}\right)}{\sum_{k=1}^{n}\exp\left({Similarity}_{i,k}\right)}$$

A new representation *Z* is obtained by weighted summation of *V* using the attention weights:

(15) $$Z_{i}=\sum_{j=1}^{n}\alpha_{i,j}V_{j}$$

### 2.3. Constructing the CNN-LSTM-SA Load Identification Model

Since the load identification problem is a time-series mapping problem rather than a direct time-series prediction, therefore, it is necessary to construct the obtained response data and load data into a time series that the network can handle. The method used here is to construct the time-series mapping dataset by cutting the time-series data X(t) with a sampling length of n into m segments with a length of k short time-series data $X_{1}(t),X_{2}(t)\ldots X_{m}(t)$, as shown in Equation (16). The time series constructed by this method can fully utilize the information of the original time domain data, ensure that the length of each time sample is consistent, and can also perform load identification with an arbitrary time-series length. At the same time, the data were normalized to the interval [−1,1] to prevent too large a gap in the order of magnitude of the data from causing the model to converge slowly or fail to converge.

(16) $$\left[\begin{array}{c} X_{1}(t)\\ X_{2}(t)\\ \vdots \\ X_{m}(t) \end{array}\right]=\left[\begin{array}{c} X_{1}(1)\cdots X_{1}(k)\\ X_{2}(1)\cdots X_{2}(k)\\ \vdots \\ X_{m}(1)\cdots X_{m}(k) \end{array}\right]=\left[\begin{array}{cccc} x(1) & x(2) & \cdots & x(k)\\ x(2) & x(3) & \cdots & x(k+1)\\ \vdots & \vdots & & \vdots \\ \vdots & \vdots & & \vdots \\ x(m) & x(m+1) & \cdots & x(n) \end{array}\right]$$

**Figure 4:**
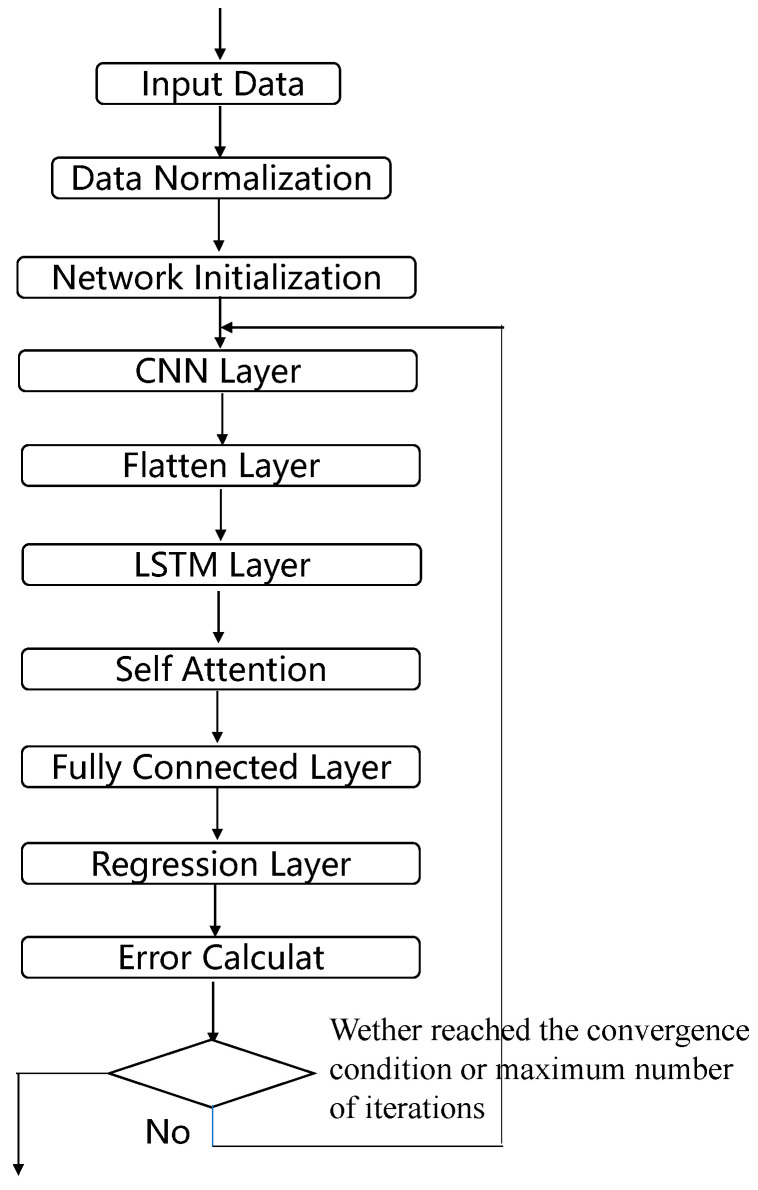
CNN-LSTM-SA network structure diagram.

The structural diagram of the constructed CNN-LSTM-SA network is shown in Figure 4, and the main steps of the model applied to the load identification work are as follows:

(1) Segment the load and response data according to the method described above and perform data normalization as well as division of the training and test sets;
(2) After initialization of the network, the response data first pass through a one-dimensional convolutional CNN network for convolutional operation and average pooling operation to extract the high-dimensional features of the data;
(3) In order to make the highly dimensional data adapt to the LSTM neural network, the data output from the CNN network need to be flattened. The flattening process consists of pulling the feature maps of each channel into one-dimensional vectors in order and then connecting the vectors of all channels;
(4) The flattened data go sequentially through the LSTM neural network, the SA network, and the fully connected network, and finally the predicted load is generated;
(5) Judge whether to terminate network training based on the error between the network’s predicted load and the actual load;
(6) The trained network performs load identification on the test set to verify the recognition effect.

The hyperparameters of this network are as follows:

(1) CNN layer: 1D convolutional kernel size, number of convolutional kernels, number of convolutional layers, and pooling size;
(2) LSTM layer: number of LSTM units, number of LSTM layers, and dropout rate;
(3) SA layer: number of attention heads, attention head dimension, and dropout rate;
(4) Hyperparameters of the network: optimizer type, learning rate, learning rate decay, batch size, max epochs, etc.

The hyperparameters of the network structure described above can be adjusted according to the complexity of the task and whether it is overfitted or not. The network hyperparameters can be determined by grid search, Bayesian optimization, and empirically. Due to the limitation of the arithmetic power, the key hyperparameters of the neural network are determined in this study by small batch grid search.

It is worth noting that the optimal value of the segmentation sequence length is affected by a variety of factors, which will not be investigated in detail here, and the length of the segmentation time sequence set in the subsequent simulation and experimental studies in this paper is 200.

## 3. Numerical Simulation Study

Thin-walled cabins are efficient structural designs widely used in aerospace, industrial production, and scientific research, and have attracted considerable attention due to their light weight, high strength, and excellent material utilization. This paper presents both numerical simulations and experiments using a thin-walled cabin as the research object, applying the proposed method for load identification.

**Figure 5:**
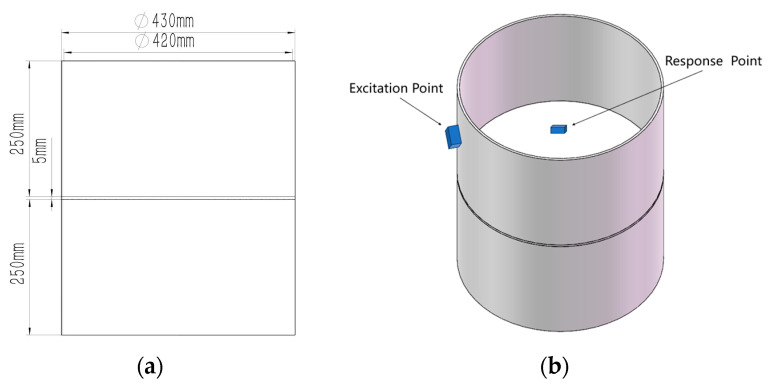
(**a**) Simulation object model; (**b**) simple harmonic excitation application position and response pickup position.

The simulation model is shown in Figure 5. The bottom face of the model is constrained with a fixed constraint and a simple harmonic excitation is applied at a point on the bulkhead wall, and the response is measured at the bulkhead bracket. Both the wall thickness of the bulkhead model and the bracket thickness inside the bulkhead are 5 mm. The model is made of aluminum alloy with a density of 2770 kg/m^3^, Young’s modulus of 71 GPa, and Poisson’s ratio of 0.33. The simulation was performed by transient analysis. The transient simulation module of ANSYS(2024R2) Mechanical software is used to calculate the simulation results. The simulation was performed using transient analysis with a time step of 0.001 s and a damping ratio of 0.03. Considering the calculation time and the accuracy of the simulation results, the mesh size is set to 2 mm.

Fifty sets of transients were analyzed for sinusoidal excitations of different frequencies and amplitudes lasting 1 s. The acceleration response at the measurement points was recorded. The sinusoidal excitation frequency ranges from 5 to 7 Hz, and a simulation calculation frequency is selected every 0.2 Hz (except 7 Hz), and transient analyses of sinusoidal excitations with amplitudes of 80 N, 110 N, 140 N, 170 N, and 200 N are performed at each frequency, respectively. The applied load time-domain data and the acquired acceleration time-domain data were divided into training (80%) and test (20%) sets for network training and load identification. To evaluate the effectiveness of the model, LSTM, CNN-LSTM, and CNN-LSTM-SA networks were selected for load identification, and their performance in load identification and ability to handle noisy data were compared.

This section uses test set error metrics to evaluate the effectiveness of the neural network load identification. The root mean square error (RMSE) and mean absolute error (MAE) are chosen as the error metrics, as defined by the following formulas:

(17) $$\mathrm{RMSE}=\sqrt{\frac{1}{n}\sum_{i=1}^{n}{\left({\hat{y}}_{i}-y_{i}\right)}^{2}}$$

(18) $$\mathrm{MAE}=\frac{1}{n}\sum_{i=1}^{n}({\hat{y}}_{i}-y_{i})\times 100\%$$

where ${\hat{y}}_{i}$ is the neural network predictive value load, $y_{i}$ is the true load of the test set, and n is the number of samples in the test set; the smaller the value of RMSE and MAE, the higher the prediction accuracy of the model.

**Figure 6:**
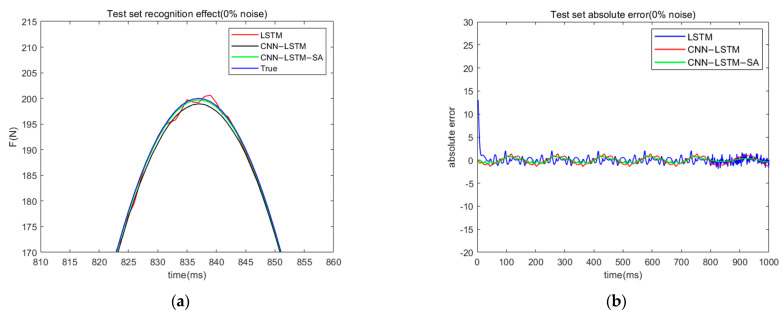
(**a**) Effectiveness of three neural network load recognition (0% noise); (**b**) absolute error of three neural network load recognition (0% noise).

The three neural networks are trained separately, and the load identification results of each network under 0% noise data are shown in Table 1. It can be seen that the load identification accuracy of CNN-LSTM-SA has a significant advantage over the other two neural networks. Figure 6 shows the recognition effect and absolute error of the three neural networks for load identification under the data without the influence of noise. From the figure, it can be seen that the CNN-LSTM-SA network can not only recognize the peak of sinusoidal excitation well, but also the absolute error is smaller than the other two networks in the whole sampling time, indicating that the CNN-LSTM-SA load identification method proposed in this paper has obvious advantages.

**Figure 7:**
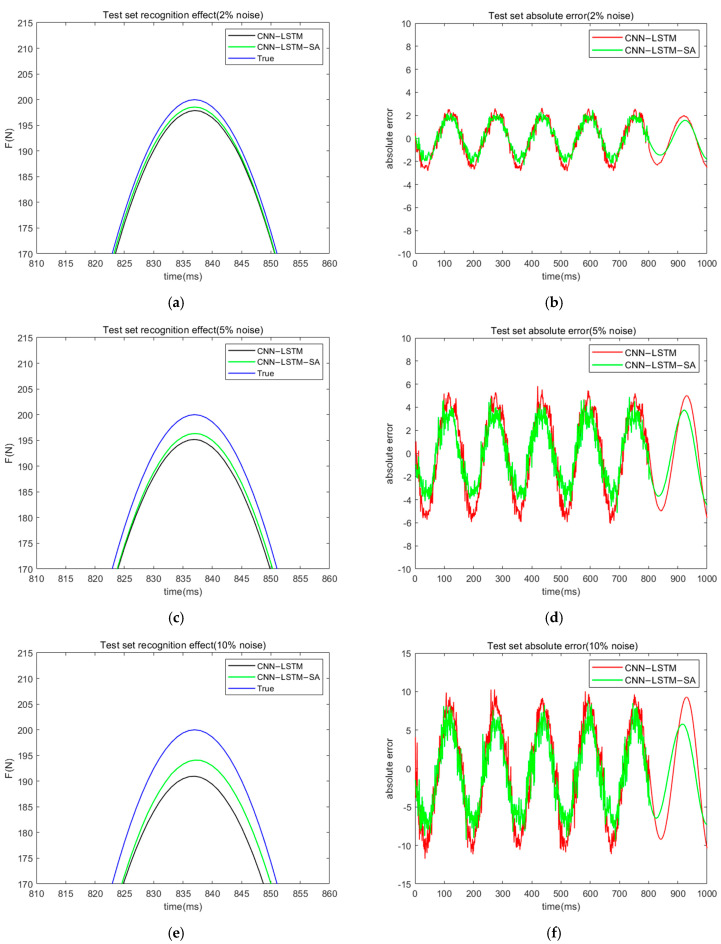

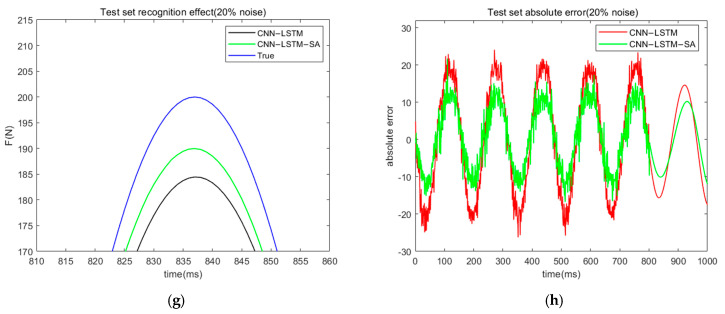
Effectiveness and absolute error (with noise) of two neural network’s load recognition. (**a**) effectiveness of two neural network’s load recognition (2% noise); (**b**) absolute error of two neural network’s load recognition (2% noise); (**c**) effectiveness of two neural network’s load recognition (5% noise); (**d**) absolute error of two neural network’s load recognition (5% noise); (**e**) effectiveness of two neural network’s load recognition (10% noise); (**f**) absolute error of two neural network’s load recognition (10% noise); (**g**) effectiveness of two neural network’s load recognition (20% noise); (**h**) absolute error of two neural network’s load recognition (20% noise).

In practical engineering problems, there is often noise interference during signal data acquisition using sensing devices, so it is necessary to verify the effectiveness of the CNN-LSTM-SA network for load recognition tasks in the presence of noisy signals. Researchers have confirmed in previous studies that CNN networks have filtering capabilities [23]; in this paper, we use the CNN-LSTM neural network as a reference to evaluate the adaptability of the CNN-LSTM-SA for load recognition tasks with noisy signals. White noise signals of 2%, 5%, 10%, and 20% are added to the simulated collected dataset, and the load recognition task is performed using the above two neural networks. Here, RMSE and MAE are still used as the indexes to evaluate the model load recognition effect, and the specific results are shown in Table 2. It can be seen that the CNN-LSTM-SA neural network has higher recognition accuracy and better noise immunity than the CNN-LSTM neural network in the load recognition work in the case of containing noise. Figure 7 shows the effect and absolute error of load recognition of both networks under different noise levels.

Based on the results of the analysis of the graphical data, it can be clearly seen that with the gradual increase in the noise level, the performance of both neural networks in the load recognition task shows a significant decrease, which is manifested by the trend of increasing their absolute error and root mean square error (RMSE). However, it is worth noting that under the influence of noise interference, the CNN-LSTM-SA neural network is still able to maintain the accuracy of load recognition well, with a relatively small increase in its error and a more stable recognition performance. In contrast, the performance of the CNN-LSTM neural network decreases more significantly, and the recognition accuracy is much lower than that of the CNN-LSTM-SA network. This indicates that the CNN-LSTM-SA network has obvious advantages in robustness and accuracy in the noisy environment.

## 4. Experimental Study

### 4.1. Test Object and Test System

**Figure 8:**
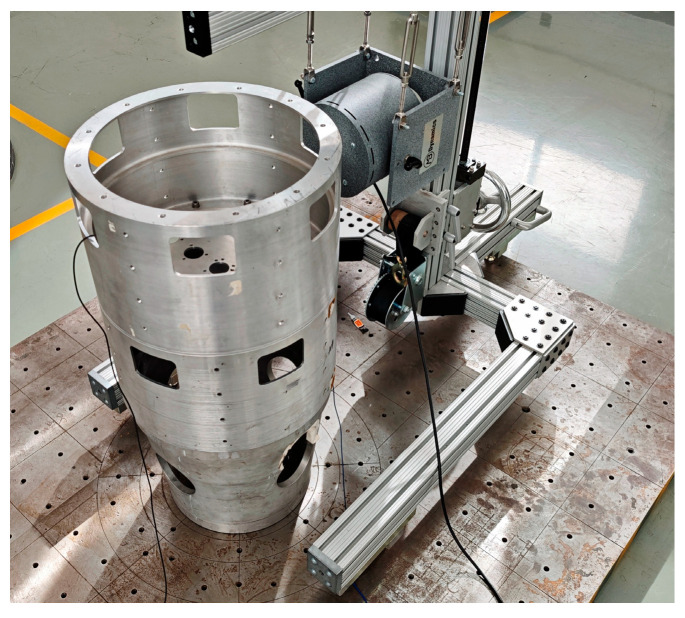
Test object.

**Figure 9:**
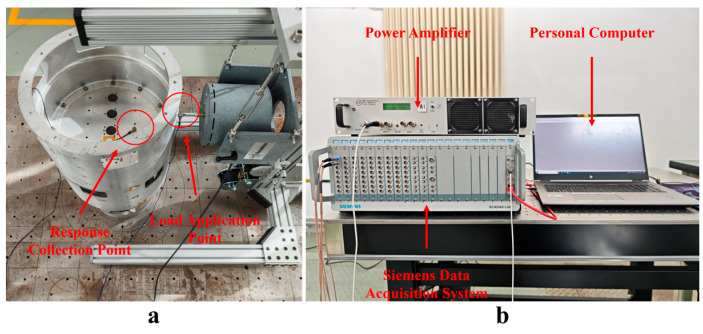
(**a**) Data acquisition location; (**b**) test system.

The bottom of the test object is a fixed constraint, as shown in Figure 8, which is used to simulate the thin-walled segment of the spacecraft; the material is aluminum alloy, and the wall thickness of the model is 5 mm. Excitation is applied by the shaker to simulate the external load of the thin-walled cabin, and the acceleration sensor is used to measure the structural response. The excitation position of the shaker and the mounting position of the acceleration sensor are shown in Figure 9a, and the test system consists of a personal computer, a power amplifier, and a data acquisition system, as shown in Figure 9b. In order to reflect the superiority of the load recognition method proposed in this paper, two load recognition methods, an LSTM neural network and a CNN-LSTM neural network, are still used for comparison, and the evaluation indexes are the RMSE, MAE, and coefficient of determination R2, where R2 is used to evaluate the fitting effect of the regression model, and the closer the value is to 1, the better the model fitting effect.

### 4.2. Time-Domain Identification of Sinusoidal Load

The experimental study of sinusoidal load identification is first carried out, and the sampling frequency of the time-series data is 6400 Hz. In this paper, 30 sets of sinusoidal excitation information with different frequencies and amplitudes and the corresponding structural acceleration responses are collected. A total of 25 sets were used to train the neural network load recognition model, and 5 test sets were used to evaluate the load recognition effectiveness of the model. The sinusoidal excitation frequency ranges from 50 to 100 Hz, and an experimental frequency (including 50 Hz) is selected every 10 Hz; data acquisition is performed at each frequency with amplitudes of 1 N, 1.5 N, 2 N, 2.5 N, and 3 N, respectively.

**Figure 10:**
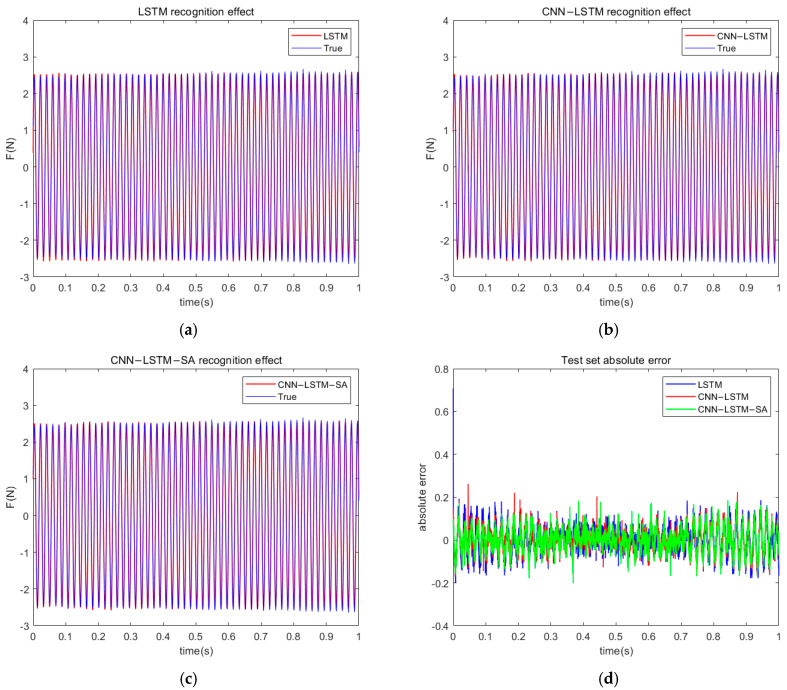
(**a**) LSTM sinusoidal load recognition effect; (**b**) CNN-LSTM sinusoidal load recognition effect; (**c**) CNN-LSTM-SA sinusoidal load recognition effect; (**d**) absolute errors in sinusoidal load recognition by three neural networks.

The sinusoidal time-domain load recognition effect of the model is shown in Figure 10a–c. From the figure, it can be observed that all three neural networks exhibit high accuracy for sinusoidal load identification. Specifically, Figure 10d shows the absolute errors of the three networks, where the CNN-LSTM-SA neural network has significantly lower errors than the other two networks in the time domain of the test set. This indicates that this network outperforms the other two models in the sinusoidal load identification task. Therefore, the CNN-LSTM-SA neural network used in this study has more superior accuracy and robustness in the sinusoidal load identification task. The model evaluation indices are shown in Table 3; compared with the other two neural networks, the neural network load identification method used in this paper has a smaller RMSE and MAE. This indicates that the method has excellent load recognition performance. Meanwhile, the R2 value of the proposed network in this paper is closest to 1, indicating that the method used in this paper has the best fitting effect among the three neural networks.

### 4.3. Time-Domain Identification of Random Load

**Figure 11:**
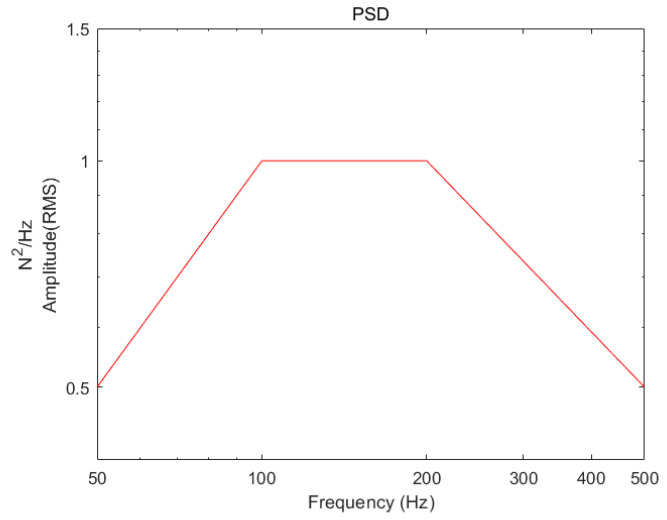
PSD for random vibration experiments.

Since the structure is often subjected to not only stable sinusoidal loads but also random loads under real working conditions, this paper also applies the proposed method to the time-domain load identification of random vibration tests to verify the generalizability of the method. Random signals are commonly described by power spectral density (PSD). In the experimental part of this study, 30 sets of time-domain excitation force and acceleration response information are collected under the same PSD with a sampling frequency of 12,800 Hz. A total of 25 of these sets are used as a training set to train the neural network load identification model, and 5 sets are used as a test set to evaluate the load identification effect of the model. The evaluation metrics used here are the same as those used for sinusoidal excitation identification. The PSD for the experimental setup is shown in Figure 11.

**Figure 12:**
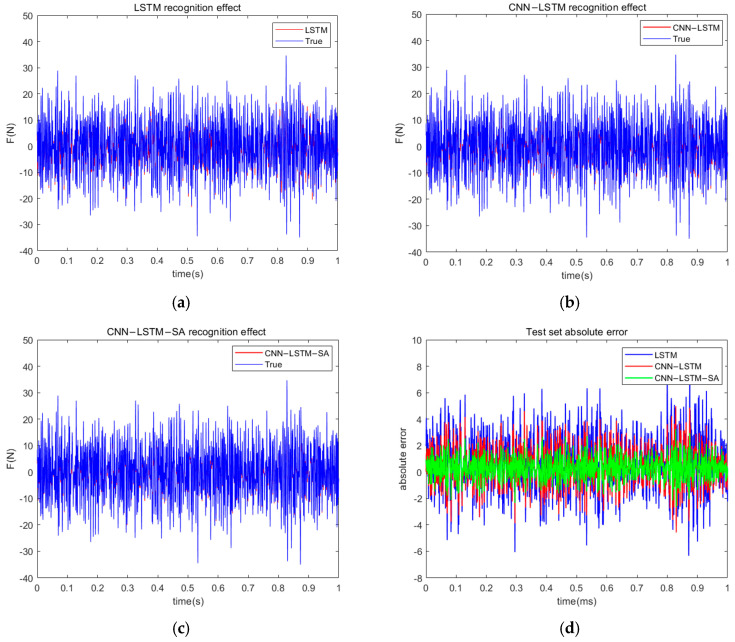
(**a**) LSTM random load recognition effect; (**b**) CNN-LSTM random load recognition effect; (**c**) CNN-LSTM-SA random load recognition effect; (**d**) absolute errors in random load recognition by three neural networks.

The random time-domain load recognition effect of the model is shown in Figure 12a–c, from which it can be seen that the three kinds of neural networks for random time-domain load have this good recognition effect. But the CNN-LSTM-SA network still has this certain advantage, which can be proved from the absolute error diagram; the absolute error diagram of the three kinds of neural networks is shown in Figure 12d. The model evaluation indices are shown in Table 4. It can be seen that, compared with the other two neural networks, the RMSE and MAE of the neural network load identification method used in this paper are smaller, which indicates that this method has an excellent load identification effect. At the same time, the R2 value of this network is closest to 1, which indicates that the method used in this paper has the best fitting effect among the three neural networks, and it also indicates that the neural network model used in this paper has the best result for recognizing the time-domain information of random loads. In summary, the neural network model used in this paper has good applicability for the time-domain information recognition of random loads.

It can be seen from the above study that under the experimental data, the load identification effect of the neural network decreases compared to the simulation data. This is mainly due to the fact that the experimental data are not only disturbed by white noise but also by abnormal time-series data and other uncertainties. Nevertheless, the neural network model proposed in this paper still shows good identification ability, which fully proves its adaptability and effectiveness in complex environments. On the other hand, the number of training parameters for the model increases significantly due to the mixing of multiple network structures. In addition, under high-frequency excitation, a higher sampling frequency is required to ensure sufficient accuracy, which also leads to an increase in the size of the dataset. The combination of these factors increases the cost of network training.

## 5. Conclusions

This paper proposes a time-domain load identification method based on a CNN-LSTM-SA neural network. The method takes segmented time-series data as input and combines the advantages of the three networks to achieve higher recognition accuracy and robustness. The main conclusions are the following:

(1) Simulation results show that for sinusoidal load identification, the CNN-LSTM-SA network has obvious advantages in terms of recognition accuracy and noise immunity. The RMSE and MAE are 0.47 and 0.53 under 0% noise and 8.8 and 8.5 under 20% noise, respectively;
(2) The experimental results show that the CNN-LSTM-SA network achieves high identification accuracies in both sinusoidal and random load identification tasks (RMSE of 0.08 and 0.83; R^2^ of 0.98 and 0.93, respectively);
(3) The CNN-LSTM-SA-based load identification method provides researchers with a tool with higher accuracy and noise immunity, as well as a reliable method for structural health monitoring and optimal design.

## Figures and Tables

**Table 1:** Neural network load recognition effect (0% noise).

	RMSE	MAE
LSTM	1.10	0.61
CNN-LSTM	0.74	0.55
CNN-LSTM-SA	0.47	0.53

**Table 2:** Neural network load recognition effect (with noise).

Noise Level	2% Noise		5% Noise		10% Noise		20% Noise	
	RMSE	MAE	RMSE	MAE	RMSE	MAE	RMSE	MAE
CNN-LSTM	1.62	1.27	3.55	3.18	6.43	5.10	13.89	12.73
CNN-LSTM-SA	1.29	1.13	2.58	2.86	4.76	4.45	8.47	10.83

**Table 3:** Effect of time-domain identification of sinusoidal loads.

	RMSE	MAE	R^2^
LSTM	0.12	0.14	0.97
CNN-LSTM	0.09	0.11	0.97
CNN-LSTM-SA	0.08	0.10	0.98

**Table 4:** Random load time-domain recognition effect.

	RMSE	MAE	R^2^
LSTM	2.07	3.42	0.88
CNN-LSTM	1.39	2.57	0.90
CNN-LSTM-SA	0.83	1.69	0.93

## Data Availability

The data provided in this study can be provided at the request of the corresponding author. The research data presented in the paper consist of simulation and experimental results. We have provided the necessary simulation settings and experimental setups within this paper to enable scholars to replicate our results. Therefore, it is not necessary for us to specifically upload the data. Additionally, we have not uploaded the data from this paper to any public datasets. However, if there is a need, we are willing to provide the data upon request.
